# Causes of Emergency Department Visits Among End-Stage Kidney Disease Patients on Maintenance Hemodialysis in Pakistan: A Single-Center Study

**DOI:** 10.7759/cureus.33004

**Published:** 2022-12-27

**Authors:** Farah Mujtaba, Aasim Ahmad, Murtaza Dhrolia, Ruqaya Qureshi, Kiran Nasir

**Affiliations:** 1 Nephrology, The Kidney Centre Postgraduate Training Institute, Karachi, PAK

**Keywords:** end-stage kidney disease, infection-related complications, electrolyte abnormalities, hemodialysis, emergency department

## Abstract

Background

In this study, we aimed to determine the causes of emergency department (ED) visits by end-stage kidney disease (ESKD) patients on maintenance hemodialysis (MHD) in Karachi, Pakistan.

Methodology

We conducted a cross-sectional study that included 194 visits of ESKD patients on MHD aged ≥18 years of both genders presenting at the ED of The Kidney Centre Postgraduate Training Institute, Karachi, Pakistan between February 2021 and May 2021. The study investigated the causes behind ED visits. Frequencies were calculated for categorical variables, and a bar graph was used for the graphical representation of the causes.

Results

In total, 194 visits included 151 patients of whom 88 (58.3%) were males while 63 (41.7%) were females, with a mean age of 51.68 ± 15.8 years. The most common comorbidity among the ED visits was hypertension 182 (93.8%). The majority of the visits 129 (66.5%) were undergoing regular three dialysis sessions per week, 101 (52.1%) were registered for MHD at our Institute, and 69.1% of visits reported arteriovenous fistula (AVF) as the current access for hemodialysis. Around 111 (57.2%) of the visits had infection-related complications, followed by electrolyte abnormalities 74 (38.1%), cardiovascular 53 (27.3%), and pulmonary complications 41 (21.1%). Overall, 19 (9.8%), 16 (8.2%), and 14 (7.2%) patients reported access-related, neurological, and gastrointestinal complications, respectively.

Conclusions

Infection-related complications are a significant cause of ED visits among ESKD patients, followed by electrolyte abnormalities and systemic complications, many of which are related to the existing comorbid conditions. Risk identification of preventable causes and surveillance of existing comorbidities would help mitigate ED visits among ESKD patients.

## Introduction

Chronic kidney disease (CKD) is recognized worldwide as an important cause of death and loss of disability-adjusted life years (DALYs). Around one million deaths are attributed to CKD [1,2]. The incidence, prevalence, and progression of CKD vary among countries with a worldwide prevalence of 13.4% [3], increasing significantly in densely populated South Asian countries, with Pakistan being in the upper range at 23.3% [4].

CKD classification has evolved over time, and, currently, stage 3 CKD is defined as decreased kidney function with a glomerular filtration rate (GFR) of less than 60 mL/minute/1.73 m^2^ [5]. There are different stages of CKD, with stage 5 being the last stage at which GFR declines to less than 15 mL/minute/1.73 m^2^. At this point, patients are diagnosed with end-stage kidney disease (ESKD) and require renal replacement therapy consisting of either costly dialysis or a kidney transplant to survive.

Compared to other chronic conditions, ESKD patients have a high rate of developing subacute, acute, and life-threatening complications that account for their frequent emergency department (ED) visits and hospitalizations [6]. There are several reasons for these recurrent ED visits, with the most common being access-related complications, sepsis, electrolyte imbalance, and fluid overload with cardiovascular and pulmonary complications [7]. In a large retrospective study conducted in the United States, it was found that ESKD patients use the ED at six times higher rate than the national mean rate for US adults. The study also reported that the admission rate from ED was four-fold higher than the national mean rate [7]. Most of these visits were due to preventable causes and modifiable risk factors.

With an enormous burden of ESKD patients in Pakistan, to our knowledge, no study has been conducted yet in our region to evaluate the causes of ED visits among these patients. Our study aims to identify a broad range of causes accounting for these visits. By assessing these causes, this study would help to identify at-risk patients, reform policies, allocate financial resources, and improve patient education and care regarding preventable causes. These measures can help to decrease the huge influx of these patients in the ED.

## Materials and methods

Study design, setting, and participants

We conducted a facility-based, observational, cross-sectional study using a non-probability sampling technique to consecutively recruit 194 patients attending the ED of The Kidney Centre Postgraduate Training Institute (TKC PGTI) between February 2021 and May 2021. All patients of either gender aged ≥18 years who were on maintenance hemodialysis (MHD) were included. Patients coming to the ED only for scheduled dialysis and those who were on peritoneal dialysis were excluded from the study.

Sample size estimation

The required sample size was calculated as 185 [7] (using the Open Epi Version 3.01) keeping a margin of error of 1%, a confidence level of 99%, a design effect of 1, and inflated to adjust for non-response and refusal.

Study procedure

Informed written consent was taken by a doctor. After studying the history, examination, and investigations done as part of the ED protocol, the cause of the ED visit was determined and recorded in the Proforma.

Study tool and variables

A structured Proforma was developed based on previous literature including demographic variables such as age, sex, and comorbidities. The study tool also comprised hemodialysis characteristics (duration of dialysis dependence, number of dialysis sessions per week, and in-center dialysis at TKC PGTI or outside) and access-related characteristics (current access and site of catheter). The Proforma included the causes of ED visits. The main categories of the complications accounting for ED visits were electrolyte disorders and infection-related, access-related, cardiovascular, pulmonary, neurological, and gastrointestinal complications. Each complication had a different subcategory of causes. The end outcome from the ED was also recorded based on evaluation during the ED visit, i.e., admitted, discharged, referred, and left against medical advice (LAMA) or discharge on request (DOR).

Statistical methods

Data were analyzed using SPSS version 21 (IBM Corp., Armonk, NY, USA) after cleaning and coding the data. Frequencies were calculated for each category of complications and subcategories of causes from the total number of visits. Many visits had causes falling into more than one category or subcategory. The frequency of each category was reported as a bar graph for better visual presentation, while the frequency of subcategories was presented as tables.

Ethical consideration

Ethical approval for the study was obtained from the Ethical Review Committee of TKC PGTI (reference number: 111-NEPH-122020 dated January 4, 2021).

## Results

A total of 200 visits that fulfilled the inclusion criteria were asked to participate, of which 194 agreed to participate, giving a response rate of around 97%. The non-responses were due to the severity of the illness because of which patients or attendants were unable to participate. In total, 194 visits included 151 patients, of whom 115 patients had a single visit to the ED during the study period, 30 patients had two visits, five patients had three visits, and one patient had four visits.

Demographic characteristics and comorbidities

The mean age of 151 patients was 51.68 ± 15.8 years, with a minimum of 19 and a maximum of 89 years. Of these 151 patients, 88 (58.3%) were males. The major comorbidities among the 194 visits were hypertension 182 (93.8%), followed by diabetes mellitus 93(47.5%), and ischemic heart disease 54 (27.8%) (Table 1).

**Table 1:** Demographic and clinical characteristics of Emergency Department visits by end-stage kidney disease patients in Karachi, Pakistan (n = 194). TKC PGTI = The Kidney Centre Postgraduate Training Institute; AVF = arteriovenous fistula; AVG = arteriovenous graft

Characteristics	n (%)
Gender (n = 151)	
Male	88 (58.3)
Female	63 (41.7)
Comorbidities	
Hypertension	182 (93.8)
Diabetes mellitus	93 (47.5)
Ischemic heart disease	54 (27.8)
Hepatitis C	16 (8.2)
Cardiovascular disease	11 (5.7)
Hepatitis B	4 (2.1)
Post-transplant	3 (1.5)
Duration of dialysis dependence	
<3 months	32 (16.5)
4–6 months	24 (12.4)
7 months to 1 year	32 (16.5)
2–3 years	56 (28.9)
4–5 years	21 (10.8)
6–10 years	20 (10.3)
>10 years	9 (4.6)
Number of dialysis sessions per week	
One session	3 (1.5)
Two sessions	62 (32.0)
Three sessions	129 (66.5)
In-center dialysis at TKC PGTI	
Yes	101 (52.1)
No	93 (47.9)
Current access	
AVF	134 (69.1)
AVG	2 (1.0)
Tunneled catheter	41 (21.1)
Non-tunneled catheter	17 (8.8)
Site of catheter (n = 58)	
Subclavian	3 (5.2)
Internal jugular	51 (87.9)
Femoral	4 (6.9)
Outcome of Emergency Department visit	
Discharged	77 (39.7)
Referred	49 (25.3)
Admitted	40 (20.6)
Leave against medical advice or discharge on request	28 (14.4)

Hemodialysis characteristics

In most of the visits, 88 (45.4%) had dialysis dependence duration of less than one year (32 (16.5%), 24 (12.4%), and 32 (16.5%) were dependent for fewer than three months, four to six months, and seven months to one year, respectively). This was followed by 56 (28.9%) who were dependent on dialysis for two to three years. Nine (4.6%) visits were dialysis dependent for >10 years. In total, 129 (66.5%) of the visits were undergoing three dialysis sessions per week of four-hour duration. Approximately half of the visits 101 (52.1%) had in-center hemodialysis at TKC PGTI (Table 1).

Access-related characteristics for hemodialysis

About 134 (69.1%) visits had an AVF as current access for hemodialysis, followed by tunneled catheter 41 (21.1%), and non-tunneled catheter 17 (8.8%). Arteriovenous graft (AVG) as access was found in only two (1%) of the visits. Out of the 58 visits that had a catheter as current access, the internal jugular was the most accessed site of the catheter in 51 (87.9%) of the visits (Table 1).

Causes of emergency department visits

Hyperkalemia 47 (24.2%) was found to be the most common electrolyte abnormality, followed by metabolic acidosis 22 (11.3%), hyponatremia 18 (9.3%), hypocalcemia 11 (5.7%), hypercalcemia 10 (5.2%), hypokalemia seven (3.6%), and the lowest being hypernatremia one (0.5%). Pulmonary edema was reported in 37 (19.1%) of the visits. The highest reported cause of cardiovascular complications was hypertensive urgency 14 (7.2%). Respiratory tract infection secondary to confirmed or suspected coronavirus disease 2019 (COVID-19) was 36 (18.6%) of visits, which was the highest among infection-related causes, followed by access-related infections 33 (17%). Out of these 33 visits with access-related infections, 32 (96.97%) visits had central venous catheters while only one (3.03%) visit had an AVF. In total, 10 (5.2%) of the total visits reported AVF/AVG thrombosis and eight (4.1%) had AVF/AVG hemorrhage. Among gastrointestinal causes, eight (4.1%) visits had gastritis. The main neurological complications were cerebrovascular accident (CVA) and encephalopathy amounting to seven (3.6%) and seven (3.6%) visits, respectively (Table 2).

**Table 2:** Causes of Emergency Department visits by end-stage kidney disease patients in Karachi, Pakistan (n = 194). ED = Emergency Department; RTI = respiratory tract infection; UTI = urinary tract infection; PVD = peripheral vascular disease; AVF = arteriovenous fistula; AVG = arteriovenous draft *Other infections include dialysate-associated infection, diabetic foot, bed sores, facial bulla, fever of unknown origin, gluteal abscess, neck abscess, and Steven-Johnson syndrome.

Causes of ED visits	n (%)
Electrolyte abnormalities	
Hyperkalemia	47 (24.2)
Metabolic acidosis	22 (11.3)
Hyponatremia	18 (9.3)
Hypocalcemia	11 (5.7)
Hypercalcemia	10 (5.2)
Hypokalemia	7 (3.6)
Hypernatremia	1 (0.5)
Pulmonary complications	
Pulmonary edema	37 (19.1)
Pleural effusion	8 (4.1)
Infection-related complications	
Respiratory tract infection (suspected or confirmed COVID-19)	36 (18.6)
Access related infection	33 (17.0)
Other infections*	17 (8.8)
Acute gastroenteritis	15 (7.7)
RTI (non-COVID-19)	12 (6.2)
UTI	8 (4.1)
Cellulitis	7 (3.6)
Cardiovascular complications	
Hypertensive urgency	14 (7.2)
Hypertensive emergency	13 (6.7)
Acute coronary syndrome	9 (4.6)
Intradialytic hypotension	8 (4.1)
Arrhythmias	7 (3.6)
Intradialytic hypertension	5 (2.6)
Heart failure	3 (1.5)
Pericardial effusion	2 (1.0)
PVD	1 (0.5)
Access related complications	
AVF/AVG thrombosis	10 (5.2)
AVF/AVG hemorrhage	8 (4.1)
Catheter dysfunction	3 (1.5)
Gastrointestinal complications	
Gastritis	8 (4.1)
Pancreatitis	3 (1.5)
Upper gastrointestinal bleed	3 (1.5)
Neurological complications	
Cerebrovascular accident	7 (3.6)
Encephalopathy	7 (3.6)
Seizures	3 (1.5)

Other complications, 10 (5.6%), which were minor and reported in frequency were two with vasovagal syncope and single with per rectal bleed, shock, septic shock, post-dialysis cramps, bleeding from the double lumen insertion site, AVF swelling, ascites, and movement disorder.

Outcomes of the emergency department visits

A total of 77 (39.7%) visits were discharged, 49 (25.3%) were referred, 40 (20.6%) were admitted, and 28 (14.4%) patients left against medical advice or were discharged on request (Table 1).

**Figure 1:**
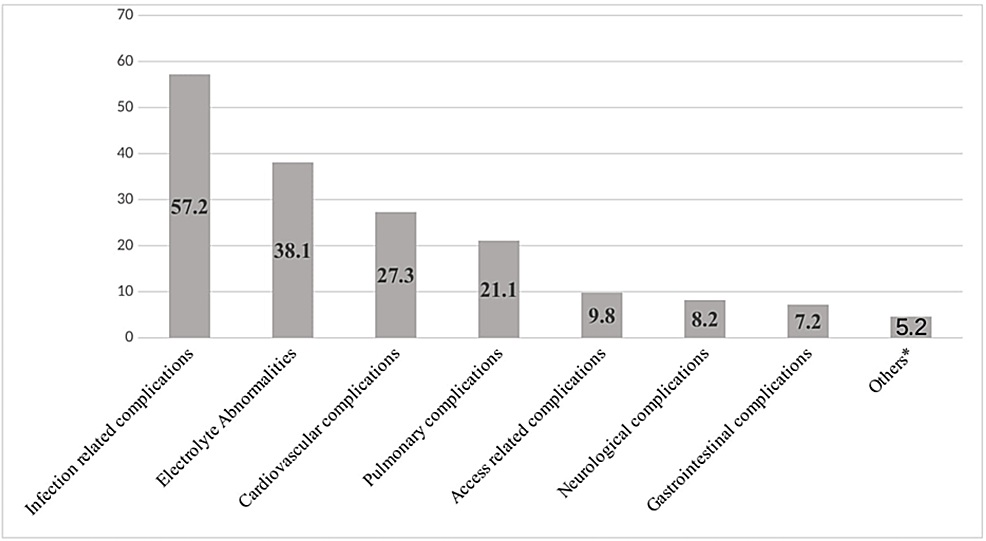
Complications among end-stage kidney disease patients attending the Emergency Department in Karachi, Pakistan (n = 194). *Other complications include per rectal bleeding, vasovagal syncope, shock, septic shock, post-dialysis cramps, bleeding from the D/L site, arteriovenous fistula swelling, ascites, and movement disorder.

All the causes of ED visits were categorized into eight groups of complications accounting for ED visits. The majority (57.2%) of the visits were infection-related, and the second most common cause was electrolyte abnormalities at 38.1%. Cardiovascular and pulmonary complications were reported in 27.3% and 21.1% of the visits, respectively. Overall, 9.8%, 8.2%, and 7.2% of the visits reported access-related, neurological, and gastrointestinal complications, respectively. The frequencies of each group of complications are described in Figure 1. Out of 74 visits with electrolyte abnormalities, two (2.7%) were on once-weekly dialysis, 30 (40.5%) were on twice-weekly dialysis, and 42 (56.8%) were on thrice-weekly dialysis sessions. In total, 41 visits had pulmonary complications, one (2.4%) was on once-weekly dialysis, 17 (41.5%) were on twice-weekly dialysis, and 23 (56.1%) were on thrice-weekly dialysis sessions.

## Discussion

In this study, we have analyzed the characteristics of ED visits by ESKD patients on MHD. Infection-related complications were the leading cause of ED visits, followed by electrolyte abnormalities.

The hospital setting where this study was conducted is a tertiary care kidney hospital where adult and pediatric patients with nephrology and urology conditions visit. The total number of visits to the ED during the study period was in 2008, of which 200 visits fulfilling the inclusion and exclusion criteria were selected. Of these, 194 agreed to participate in the study.

Around 88 (58.3%) of the visits were male while 63 (41.7%) were female. This male predominance among ESKD patients has been reported in studies from the United States and Japan as well [8]. Further research on gender interaction with ESKD is needed. The comorbid conditions commonly reported were hypertension 182 (93.8%), diabetes mellitus 93 (47.5%), and ischemic heart disease 54 (27.8%). Because diabetes and hypertension are the most common causes of CKD, they were found in many of our patients. Cardiovascular disease alone has a strong association with ESKD and is a significant cause of mortality among them [9]. With advancing age and multiple comorbidities, ESKD patients can be expected to visit the ED frequently.

The majority 88 (45.4%) were on dialysis for less than one year. In a study done in the United States, more than half of the ESKD patients who visited the ED had their MHD started within a year similar to our study [7]. The common reasons for such frequent visits of newly diagnosed ESKD patients were complications related to temporary access, inadequate dialysis, and fluid overload due to lack of dry weight adjustment. In our study, 129 (66.5%) visits were having standard thrice-weekly MHD while 62 (32%) were on twice-weekly sessions.

About 136 (70.1%) of our visits had permanent access. The remaining 58 (29.9%) visits had temporary access, with 41 (21.1%) having tunneled catheters, and 17 (8.8%) having non-tunneled catheters. As non-tunneled catheters are placed for a short duration due to the risk of complications, it was reported in only a few visits. Temporary catheters were placed either due to permanent access dysfunction, failure, or impending maturation.

Most visits were found to have infection-related complications, with a frequency of 111 (57.2%). Because our study was conducted at the time of the COVID-19 pandemic, 36 (18.6%) of the visits were suspected or confirmed cases of COVID-19. COVID-19 suspicion was based on clinical, laboratory, and radiological findings. Symptoms such as shortness of breath, fever, cough, flu, body aches, loss of taste or smell, diarrhea, or sore throat; laboratory investigations such as lymphopenia, thrombocytopenia, leucopenia, or hypoxia on arterial blood gas report; and radiological investigations showing peripheral and basal ground-glass opacity or consolidation on chest X-ray were considered suspicious of COVID-19. Rapid antigen test for COVID-19 was not available in our ED at the time of this study. A comparative study on the first and second wave of COVID-19 among MHD patients conducted at our institute found 11.8% and 10.9% confirmed cases of COVID-19 during the first and second wave, respectively [10].

Access-related infections in 33 (17.0%) visits were the second most common infection reported. Overall, 32 out of these 33 visits with access-related infections had a central venous catheter. Our analysis is supported by similar findings in a study conducted before the COVID-19 pandemic which showed that catheter-related infection was the most common cause of admission to the ED among ESKD patients [7]. A study on ED utilization patterns by ESKD patients showed that the presence of hemodialysis catheters was associated with 22% higher odds of ED presentation [6]. Catheter-associated bacteremia has a significant contribution to morbidity and mortality among patients with ESKD [11]. The factors associated with bloodstream infections include the duration of the catheter, previous catheter-associated bacteremia, specific site catheter, e.g., femoral or left-sided internal jugular vein, immunosuppressive disorders, older age, diabetes, and cardiovascular diseases [11,12]. Other reported infections are acute gastroenteritis, non-COVID-19 respiratory tract infection, urinary tract infection, cellulitis, and some other causes, as mentioned in Table 2.

Electrolyte abnormalities are commonly seen among patients with inadequate dialysis, non-adherence to dietary or fluid restrictions, and non-compliance with CKD medications. Around 74 (38.1%) visits had electrolyte abnormalities, with hyperkalemia being the most frequently 47 (24.2%) reported in a similar study as well [7]. Metabolic acidosis in 22 (11.3%) visits was the second most common electrolyte abnormality found in our study. The decreased renal excretion of hydrogen ions is responsible for metabolic acidosis in ESRD patients [13]. Moreover, inadequate dialysis, increased protein intake in the diet, and gastrointestinal loss of bicarbonate are also responsible for it [13]. The control of metabolic acidosis in ESRD patients demands the control of these causative factors. Other electrolyte abnormalities observed in our study were hyponatremia, hypocalcemia, hypercalcemia, and hypokalemia, as mentioned in Table 2. Hyponatremia and hypokalemia are often attributed to the dilutional effects of fluid overload, drug-induced, or as a complication of dialysis. However, calcium disturbances are mostly due to undertreatment or overtreatment of mineral bone disorders in ESKD patients.

Cardiovascular disease is a major cause of morbidity and mortality among ESKD patients, with a prevalence of >50%, and a relative risk of death 20 times higher than the general population [14]. Several risk factors for the development of cardiovascular disease include left ventricular hypertrophy, age, male gender, anemia, hypertension, diabetes, dyslipidemia, uremia, mineral bone disorder, and oxidative stress [14]. Cardiovascular complications were found in 53 (27.3%) visits. Around 27 (13.9%) presented with hypertensive urgency or emergency, and 13 (6.7%) were referred to the ED due to intradialytic hypertension or hypotension. A summary report of ED visits by ESKD patients developed by the University of Michigan Kidney Epidemiology and Cost Center (UM-KECC) found 4.5% admitted and 6.1% treated as an outpatient for ischemic heart disease from the ED [15]. Blood pressure-related complications are observed in ESKD patients due to non-restriction of salt and water intake, poor control of dry weight, and non-compliance with antihypertensive medications. Other cardiovascular complications noted were acute coronary syndrome, arrhythmias, and heart failure with frequencies, as mentioned in Table 2.

Pulmonary complications were found in 41 (21.1%) of the visits, mainly due to fluid overload. Hyperkalemia and fluid overload are preventable complications in ESKD patients and were a common finding in a similar study [7].

Among access-related complications, AVF/AVG thrombosis and hemorrhage were observed in 10 (5.2%) and eight (4.1%) respectively. AVG thrombosis is more common than AVF thrombosis and often requires intervention [16]. Access hemorrhage is mainly due to excessive heparinization, outflow stenosis, laceration during needling, and aneurysms or pseudoaneurysms.

Relatively fewer common causes of ED visits were neurological and gastrointestinal complications reported at 16 (8.2%) and 14 (7.2%), respectively. The main neurological complications observed were CVA, encephalopathy, and seizures. Metabolic derangements [17] and septic states often account for the development of encephalopathy and seizures. UM-KECC also found 1.8% admitted and 6.1% treated as outpatients in the ED for neurological complications among ESKD patients [15]. The risk of CVA is 5-30 times higher in CKD and ESKD patients, with a mortality rate of around 90% [18]. Important risk factors include atherosclerosis, malnutrition, inflammation, uremic toxins, use of anticoagulants, old age, diabetes, and hypertension [18]. Gastrointestinal complications observed in our study were gastritis, pancreatitis, and upper gastrointestinal bleeding. Gastritis is often seen in CKD patients due to uremia and high gastrin levels, while upper gastrointestinal bleed is mainly attributed to platelet dysfunction secondary to uremia, use of heparin, and increased risk of gastrointestinal ulcers [19].

Regarding the outcome of the visits, 77(39.7%) were discharged while 40 (20.6%) were admitted and 49 (25.3%) were referred mainly due to confirmed or suspected COVID-19 or a condition requiring multidisciplinary care. A study conducted at a tertiary care setup in the United States on ED visits by ESKD patients showed that 46.2% of the visits resulted in hospital admissions [7]. The frequency of admitted and referred visits from our ED would be 89 (45.9%), similar to the above study.

Our study has considerable strengths. It is one of its kind to identify the causes behind ED visits among ESKD patients on MHD in Pakistan. Our study has reported systemic causes as well as individual causes from these broad categories. Moreover, the research findings can be utilized to explore innovative strategies for managing complications.

Because this study was conducted during the COVID-19 pandemic, COVID-19-associated infection had the leading frequency among infection-related complications. The frequency of other causes can, however, be considered during the post-pandemic era as the frequency of each subcategory was individually calculated out of the total visits. The generalizability of the study may be limited as it was done in a specialized tertiary care renal hospital.

## Conclusions

Infection-related complications and electrolyte abnormalities were significant causes of ED visits in our study, followed by cardiovascular and pulmonary complications. The high incidence of these complications calls for the need for surveillance of CKD patients and permanent access formation by CKD stage 4. Identification of preventable causes and monitoring comorbid conditions and risk factors along with standard dialysis treatment would help to decrease ED visits among ESKD patients.
